# Supplementary material for: Minimalistic In Vitro Culture to Drive Human Naive B Cell Differentiation into Antibody-Secreting Cells
Source: Cells. 2021 May 12;10(5):1183. doi: 10.3390/cells10051183 (PMC8151070; doi:10.3390/cells10051183)
Supplement: Supplementary file 1 [file cells-10-01183-s001.zip › cells-1134177-supplementary.pdf]

Table S1. Primer sequences

Genes		Sequence (5′-3′)
18S-rRNA	Forward	CGGCTACCACATCCAAGGAA
	Reverse	GCTGGAATTACCGCGGCT
AICDA	Forward	GACTTTGGTTATCTTCGCAATAAGA
	Reverse	GGTCCCAGTCCGAGATGTA
PRDM1	Forward	AACGTGTGGGTACGACCTTG
	Reverse	ATTTTCATGGTCCCCTTGGT
XBP1	Forward	CCGCAGCACTCAGACTACG
	Reverse	TGCCCAACAGGATATCAGACT
PAX5	Forward	ACGCTGACAGGGATGGTG
	Reverse	CCTCCAGGAGTCGTTGTACG
BACH2	Forward	TTGCCTGAGGAGGTCACAG
	Reverse	ACAGGCCATCCTCACTGTTC
IRF8	Forward	AACTGGACATTTCCGAGCCA
	Reverse	AATCGTCCACAGAAGGCTCC

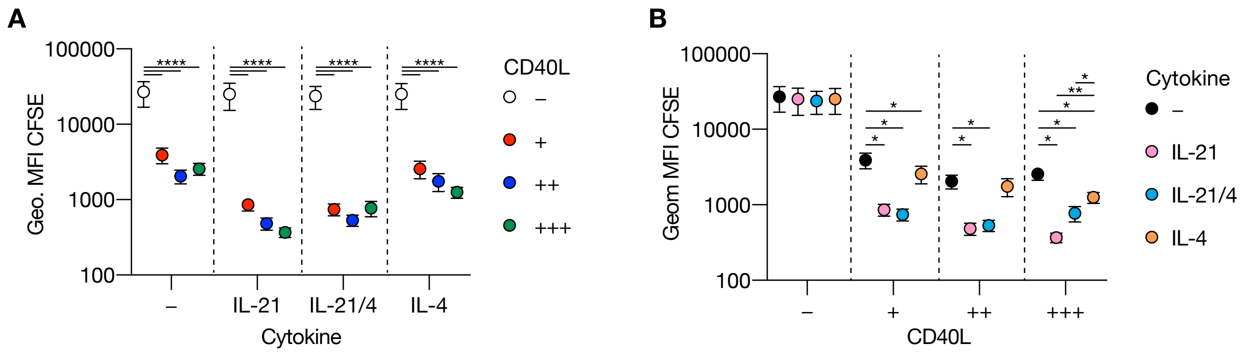

Figure S1. Level of CD40L expression affects expansion of six day stimulated naive B cells. (**A-B**) Human naive B cells were cultured on WT or CD40L-expressing 3T3 cells (as in **Figure. 1A**) with or without IL-21 and/or IL-4 for 6 days. Geometric mean fluorescent intensity (Geo. MFI) of the proliferation-dye CFSE of live $CD19^+$ cells was measured using flow cytometry. Data are shown as mean ± SEM (n=6 independent experiments). Single experiments were conducted in triplicate. Data were analyzed by a two-way ANOVA followed by Tukey's multiple comparison test. * $P \leq 0.05$, ** $P \leq 0.01$, *** $P \leq 0.001$, **** $P \leq 0.0001$.

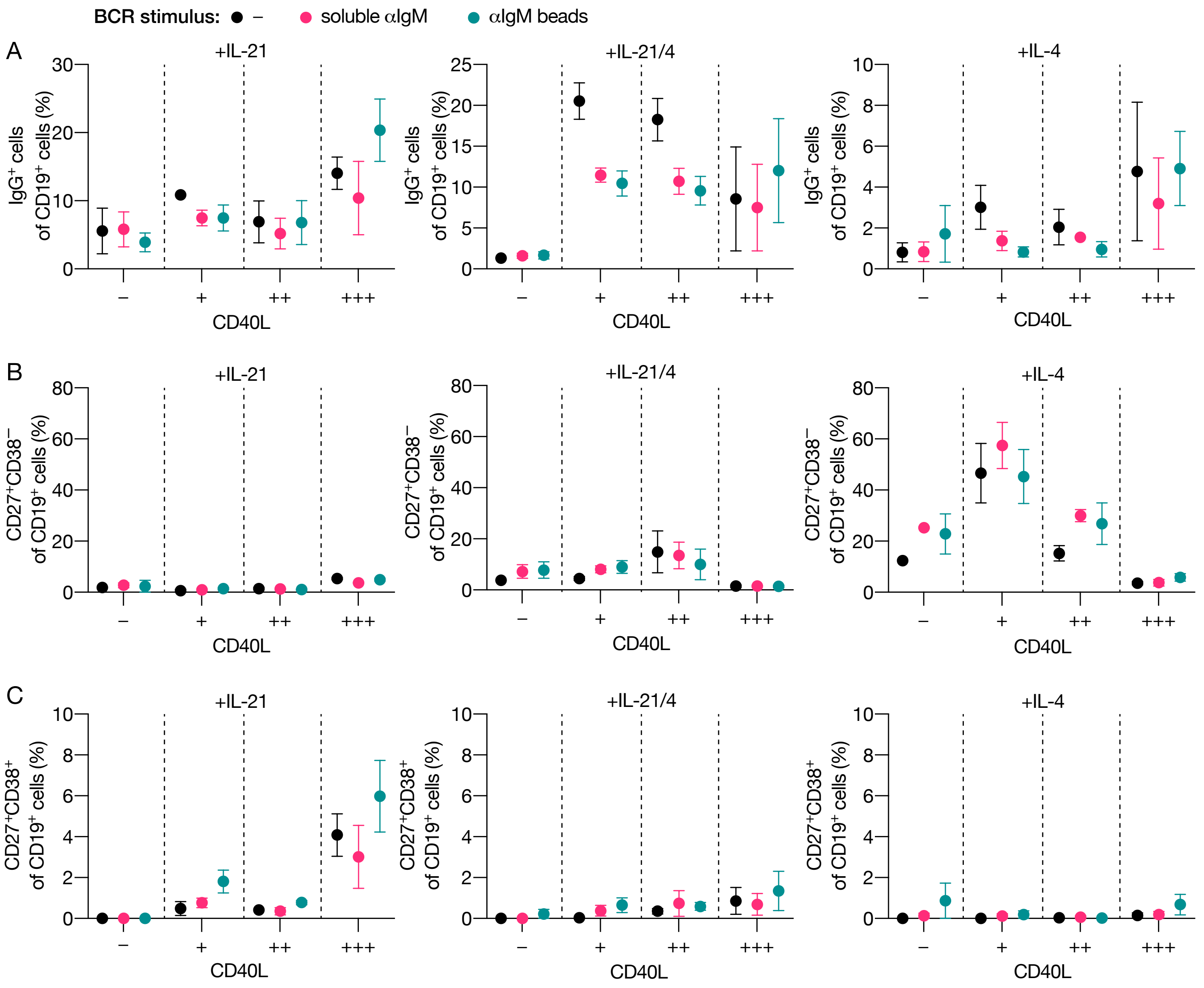

Figure S2. BCR ligation does not affect human naive B cell differentiation upon CD40 co-stimulation. (**A-C**) Human naive B cells were cultured on 3T3 cells either or not expressing CD40L (as in **Figure 1A**) with or without IL-21 and/or IL-4 for 6 (**A**) and 11 days (**B-C**). B cell receptors were ligated using soluble anti-IgM antibodies or anti-IgM coated polystyrene beads. (**A-C**) Frequencies of IgG^+ B cells (**A**), $CD27^+CD38^-$ cells (**B**) and $CD27^+CD38^+$ cells (**C**) were measured using flow cytometry. Data are shown as mean ± SEM (n=2 independent experiments). Single experiments were conducted in triplicate. Data were analyzed by a two-way ANOVA followed by Tukey's multiple comparison test.

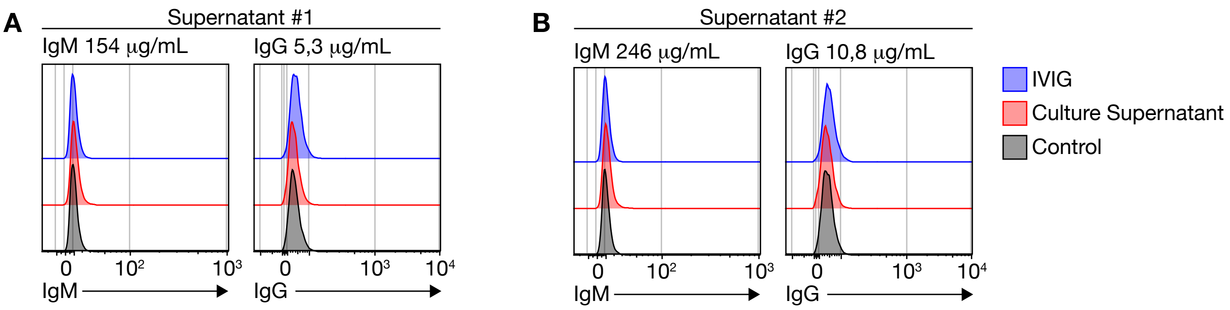

Figure S3. Secreted antibodies do not recognize 3T3 cells. Culture supernatants that contain vast amounts of secreted antibodies of unswitched (IgM) and class-switched (IgG) isotypes were incubated with 3T3 cells in suspension. Intravenous immunoglobulin (IVIG) that contains a wide range of antibodies was used to determine cross-reactivity.

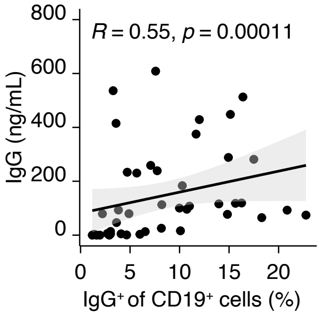

Figure S4. Moderate correlation between the frequency of IgG cells and secreted IgG. Human naive B cells were cultured on 3T3 cells expressing varying levels of CD40L with or without IL-21 and/or IL-4 and analyzed for class switching to surface IgG after 6 days and cumulative secretion of IgG after 11 days. Spearman's rank correlation coefficient was used to describe the association between frequency of IgG cells and secreted IgG.

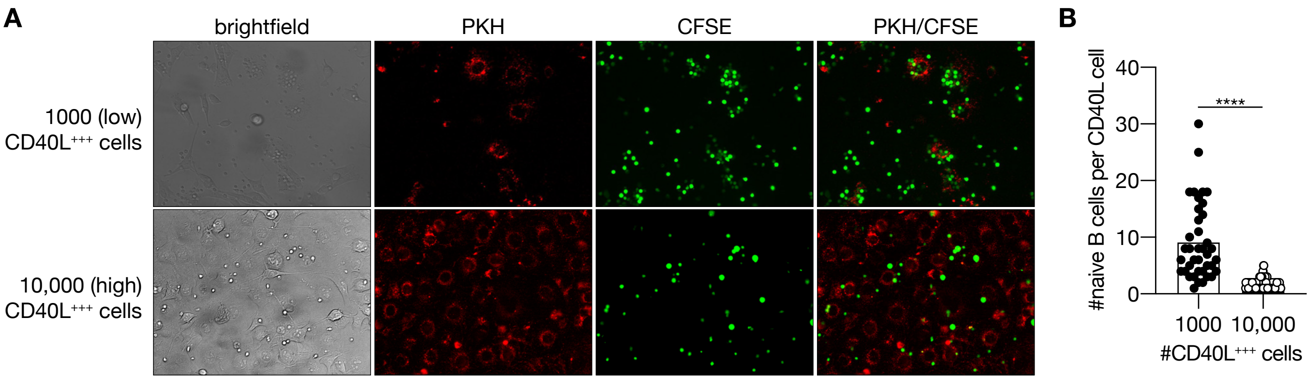

Figure S5. Naive B cells compete for CD40L-expressing cells. (**A**) CFSE-labeled naive B cells (green) cultured on 1000 (supplemented with 9000 WT 3T3 cells) or 10,000 CD40L^{+++}-expressing PKH-labeled 3T3 cells (red). Cultures were imaged every 10 minutes during the time course of 48 hours. Representative images are shown of conditions at 9 hours after start of co-culture (**A**) and quantification (**B**) was performed after 4, 9, 20 and 38 hours. Data were analyzed by an unpaired t test. **** $P \leq 0.0001$.

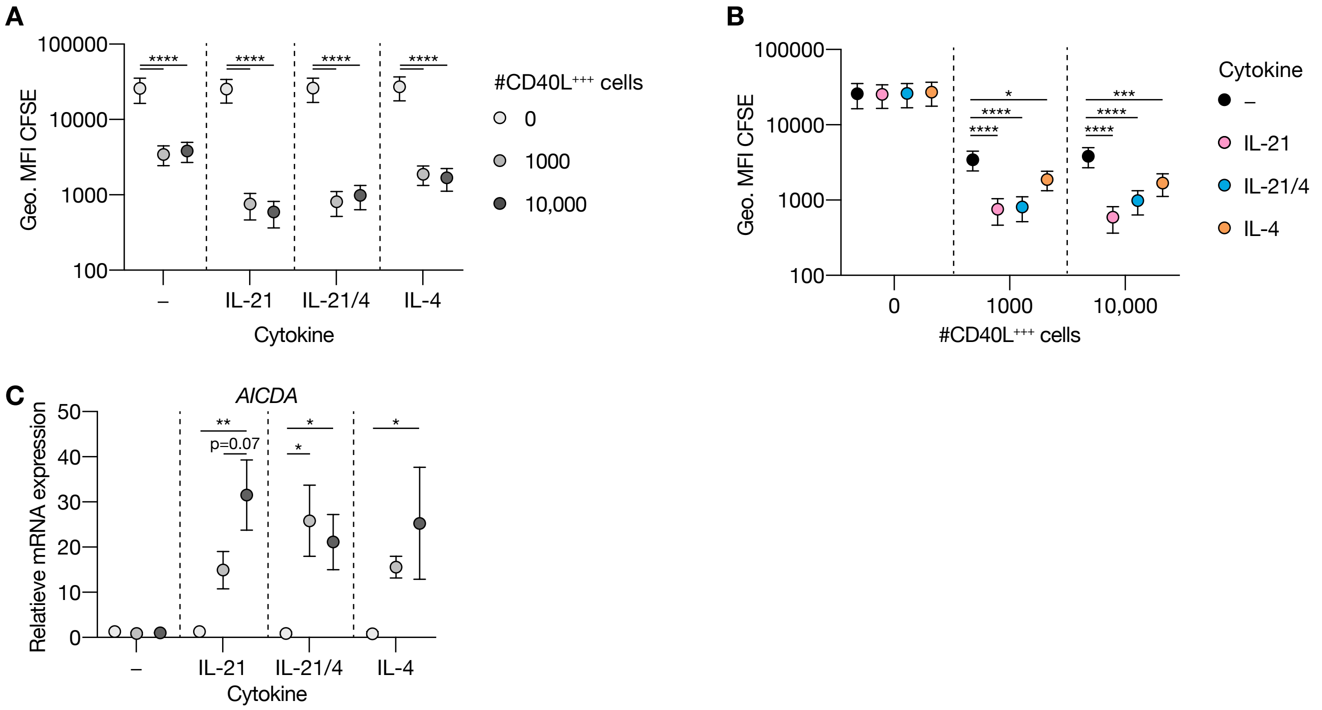

Figure S6. The number of CD40L-expressing cells affects naive B cell expansion and *AICDA* mRNA expression. (**A-B**) Human naive B cells were cultured on a ratio WT: $CD40L^{+++}$-expressing 3T3 cells (10:0, 9:1 or 0:10) with or without IL-21 and/or IL-4 for 6 days (n=7). Geometric mean fluorescent intensity (Geo. MFI) of the proliferation-dye CFSE of live $CD19^{+}$ cells was measured using flow cytometry. (**C**) *AICDA* mRNA expression after 3 days of culture on 0/1000/10,000 $CD40L^{+++}$-expressing 3T3 cells with or without cytokines (n=3). *AICDA* mRNA levels were expressed relative to expression levels in B cells stimulated for three days with 10,000 $CD40L^{+++}$-expressing 3T3 cells without cytokines. Data are shown as mean ± SEM of independent experiments. Single experiments were conducted in triplicate. Data were analyzed by a two-way ANOVA followed by Tukey's multiple comparison test. * $P \leq 0.05$, ** $P \leq 0.01$, *** $P \leq 0.001$, **** $P \leq 0.0001$.

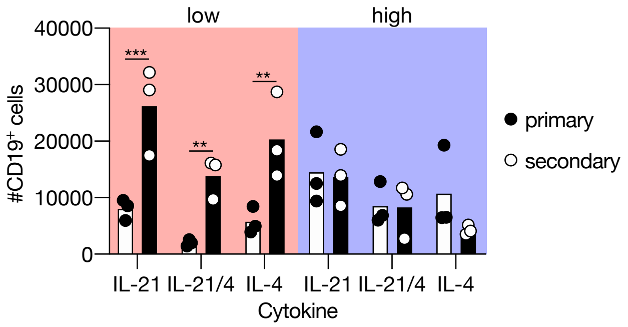

Figure S7. Re-stimulation via CD40 and Tfh cytokine signaling minimally affects number of live CD19^{+} cells. Human B cells cultured on 1000 (supplemented with 9000 WT 3T3 cells; low) or 10,000 (high) CD40L^{+++}-expressing 3T3 cells (as in **Figure 1A**) with or without IL-21 and/or IL-4 for 11 days (primary - initial only stimulation). Alternatively, primary cultures were harvested after 6 days and secondary cultures were initiated for 5 days with the same number of CD40L^{+++}-expressing 3T3 cells and similar cytokine environments as in the primary culture. The number of live CD19^{+} events were assessed 11 days in primary or secondary culture using flow cytometry. Each data point represents the mean of an individual experiment (n=3) with triplicate measurements. Mean values are represented as bars. *p*-values were calculated using multiple t-test. * $P \leq 0.05$, ** $P \leq 0.01$, *** $P \leq 0.001$.

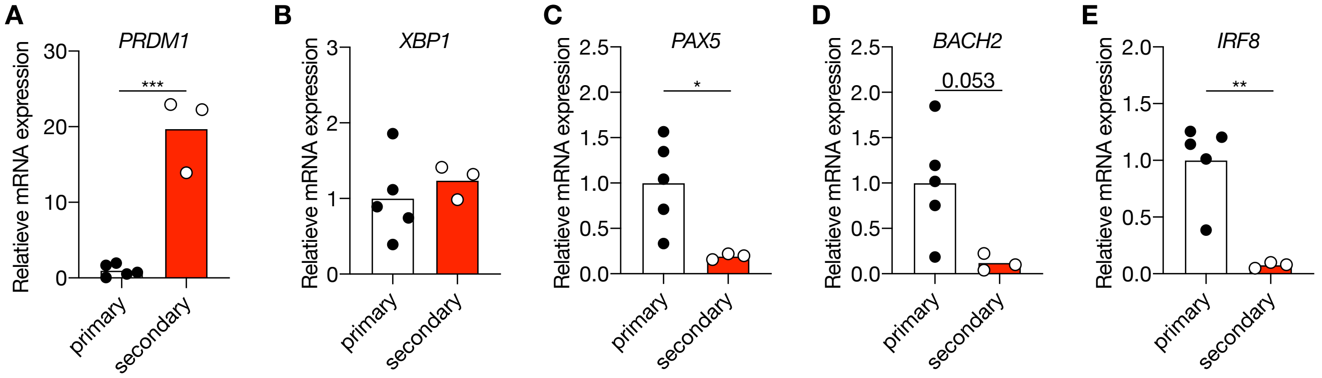

Figure S8. B cells are transcriptionally prepared before transition into the antibody-secreting cell fate. Human naive B cells were cultured on 10,000 $CD40L^{+++}$-expressing 3T3 cells (as in **Figure 1A**) and IL-21 for 9 days. Alternatively, primary cultures were harvested after 6 days and secondary cultures were initiated for 5 days with 10,000 $CD40L^{+++}$-expressing 3T3 cells and IL-21 (n=3). Subsequently, the $CD27^{-}CD38^{-}$ cell population was purified by cell sorting. (**A-E**) Expression of *PRMD1* (**A**), *XBP1* (**B**), *PAX5* (**C**), *BACH2* (**D**) and *IRF8* (**E**) mRNA in the $CD27^{-}CD38^{-}$ cell population was analyzed by qPCR relative to levels present in the $CD27^{-}$ $CD38^{-}$ cell population after 9 days in primary culture. Each data point represents the mean of an individual experiment (n=5 for primary culture and n=3 for secondary culture) with triplicate measurements. Mean values are represented as bars. *p*-values were calculated using unpaired t-test. * $P \leq 0.05$, ** $P \leq 0.01$, *** $P \leq 0.001$.

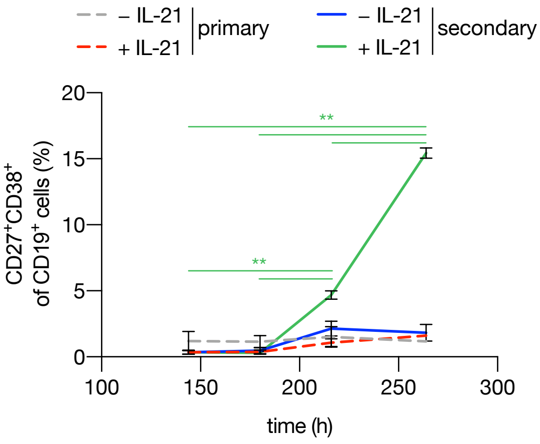

Figure S9. Re-stimulation via CD40 and IL-21 signaling efficiently promotes B cell fate transition into $CD27^{+}CD38^{+}$ antibody-secreting cells. Human naive B cells were cultured on 10,000 $CD40L^{+++}$-expressing 3T3 cells (as in **Figure 1A**) with or without IL-21 for 6 days. After 6 days, primary cultures were harvested and secondary cultures were initiated for 5 days with 10,000 $CD40L^{+++}$-expressing 3T3 cells with or without IL-21 (n=3). The frequency of $CD27^{+}CD38^{+}$ cells was analyzed at 144, 180, 216 and 264 hours (h) in primary culture (dotted lines); and 36, 72 and 120 h in secondary culture (solid lines) using flow cytometry. Data are shown as mean ± SEM (n=3 independent experiments). Single experiments were conducted in triplicate. Data were analyzed by a two-way ANOVA followed by Tukey's multiple comparison test. ** $P \leq 0.01$.
